# Unlocking Nature’s Toolbox: glutamate-inducible recombinant protein production from the *Komagatella phaffii PEPCK* promoter

**DOI:** 10.1186/s12934-024-02340-1

**Published:** 2024-02-24

**Authors:** Neetu Rajak, Trishna Dey, Yash Sharma, Vedanth Bellad, Pundi N. Rangarajan

**Affiliations:** Department of Biochemistry, Indian Institute of Science, Bangalore, 560012 India

**Keywords:** *Komagataella phaffii*, PEPCK promoter, Glutamate inducible expression system, Monosodium glutamate

## Abstract

**Background:**

*Komagataella phaffii (a.k.a. Pichia pastoris)* harbors a glutamate utilization pathway in which synthesis of glutamate dehydrogenase 2 and phosphoenolpyruvate carboxykinase (PEPCK) is induced by glutamate. Glutamate-inducible synthesis of these enzymes is regulated by Rtg1p, a cytosolic, basic helix-loop-helix protein. Here, we report food-grade monosodium glutamate (MSG)-inducible recombinant protein production from *K. phaffii PEPCK* promoter (*P*_*PEPCK*_) using green fluorescent protein (GFP) and receptor binding domain of SARS-CoV-2 virus (RBD) as model proteins.

**Results:**

*P*_*PEPCK*_*-RBD/GFP* expression cassette was integrated at two different sites in the genome to improve recombinant protein yield from *P*_*PEPCK*_. The traditional, methanol-inducible alcohol oxidase 1 promoter (*P*_*AOX1*_) was used as the benchmark. Initial studies carried out with MSG as the inducer resulted in low recombinant protein yield. A new strategy employing MSG/ethanol mixed feeding improved biomass generation as well as recombinant protein yield. Cell density of 100–120 A_600_ units/ml was achieved after 72 h of induction in shake flask cultivations, resulting in recombinant protein yield from *P*_*PEPCK*_ that is comparable or even higher than that from* P*_*AOX1*_.

**Conclusions:**

We have designed an induction medium for recombinant protein production from *K. phaffii P*_*PEPCK*_ in shake flask cultivations. It consists of 1.0% yeast extract, 2.0% peptone, 0.17% yeast nitrogen base with ammonium sulfate, 100 mM potassium phosphate (pH 6.0), 0.4 mg/L biotin, 2.0% MSG, and 2% ethanol. Substitution of ammonium sulphate with 0.5% urea is optional. Carbon source was replenished every 24 h during 72 h induction period. Under these conditions, GFP and RBD yields from *P*_*PEPCK*_ equaled and even surpassed those from *P*_*AOX1*_. Compared to the traditional methanol-inducible expression system, the inducers of glutamate-inducible expression system are non-toxic and their metabolism does not generate toxic metabolites such as formaldehyde and hydrogen peroxide. This study sets the stage for MSG-inducible, industrial scale recombinant protein production from *K. phaffii P*_*PEPCK*_ in bioreactors.

**Supplementary Information:**

The online version contains supplementary material available at 10.1186/s12934-024-02340-1.

## Background

*Komagataella phaffii*, also known as *Pichia pastoris*, can utilize amino acids such as glutamate, aspartate and proline as the sole source of carbon [1, 2]. The sequential conversion of glutamate into α-ketoglutarate, oxaloacetate and phosphoenolpyruvate is catalyzed by the cytosolic enzymes glutamate dehydrogenase 2 (GDH2), aspartate aminotransferease 2 (AAT2) and phosphoenolpyuvate carboxykinase (PEPCK), respectively [3, 4]. In *K. phaffii,* synthesis of GDH2 is induced by glutamate but not by glucose, glycerol, ethanol or acetate [4]. In contrast, PEPCK synthesis is induced by glycerol, ethanol, acetate and glutamate, with glutamate being the most efficient inducer [4]. Synthesis of GDH2 and PEPCK is also induced during carbon starvation [3]. Glutamate-inducible GDH2 and PEPCK synthesis is regulated by a cytosolic, basic, helix-loop-helix leucine zipper protein known as Rtg1p [2]. While glutamate is the inducer of GDH2 synthesis, oxaloacetate (OAA) generated during the metabolism of glutamate, ethanol and acetate is likely to be the inducer of PEPCK synthesis [4].

*K. phaffii* is a methylotrophic yeast, and the synthesis of key enzymes of the methanol utilization pathway, such as alcohol oxidase 1 (AOX1), is induced by methanol. The *AOX1* promoter (*P*_*AOXI*_) has been exploited for methanol-inducible production of thousands of recombinant proteins over the last three decades [5]. However, the highly flammable and hazardous nature of methanol necessitates safety precautions for its industrial use [5–9]. Furthermore, methanol consumption causes high heat evolution and an increased oxygen demand [10]. Hydrogen peroxide produced during methanol metabolism leads to oxidative stress, resulting in proteolytic degradation of certain recombinant proteins [11–13]. To address these issues, methanol-free, *P*_*AOXI*_-based expression systems have been developed [14–16]. Promoters of genes encoding glyceraldehyde-3-phosphate dehydrogenase (*P*_*GAPDH*_) [17], isocitrate lyase 1 (*P*_*ICL1*_) [18], formaldehyde dehydrogenase (*P*_*FLD1*_) [19], translation elongation factor 1-α (*P*_*TEF1*_) [20], phosphoglycerate kinase (*P*_*PGK1*_) [21], sorbitol dehydrogenase (*P*_*SBT1*_) [22], peroxiredoxin (*P*_*0547*_), mitochondrial aldehyde dehydrogenase (*P*_*0472*_) [23], alcohol dehydrogenase (*P*_*ADH3*_) [24], heat-shock protein 12 (*P*_*PDH*_) [25] and enzymes of thiamine biosynthesis (*P*_*THI11*_) [26] have also been used for recombinant protein expression in *K. phaffii*. The identification of GDH2 and PEPCK as glutamate-inducible enzymes and the demonstration of glutamate-inducible expression of green fluorescent protein (GFP) from the promoters of *K. phaffii GDH2* (*P*_*GDH2*_) and *PEPCK* (*P*_*PEPCK*_) led us to examine the feasibility of developing a glutamate-inducible expression system. In this study, we demonstrate food-grade monosodium glutamate (MSG)-inducible recombinant protein production from *P*_*PEPCK*_ in shake flask cultures paving the way for a glutamate-inducible *K. phaffii* expression system.

## Methods

### Media and culture conditions

A single colony of *K. phaffii* was inoculated from agar (2.0%) plates containing YPD (1.0% yeast extract, 2.0% peptone, 2.0% glucose) into YPD liquid medium and grown overnight at 30 °C in an orbital shaker at 180 rpm. Cells were washed with sterile distilled water at least twice and transferred to different minimal media containing 0.17% yeast nitrogen base (YNB, BD Biosciences, Cat: 291940) without amino acids and with 0.5% ammonium sulfate supplemented with 1.0% glutamate (YNB Glu) or 1% monosodium glutamate (YNB MSG) and 1% ethanol (YNBE). For induction of recombinant proteins in *K. phaffi*, buffered medium containing 1.0% yeast extract, 2.0% peptone, 0.17% YNB without amino acids and with 0.5% ammonium sulfate, 100 mM potassium phosphate (pH 6.0), 0.4 mg/L biotin and 2.0% methanol (BMMY), 2.0% MSG (BMYMSG), or 2% ethanol (BMEY) was added. For induction in INDI-1 medium, primary culture was transferred to buffered medium containing 1.0% yeast extract, 2.0% peptone, 0.17% YNB without amino acids and with 0.5% ammonium sulfate, 100 mM potassium phosphate (pH 6.0), 0.4 mg/L biotin, 2.0% MSG, and 2% ethanol. The induction was carried out in a shake flask (culture to flask volume ratio 1:10) at 30 °C in an orbital shaker at 180 rpm. It should be noted that buffered modified media containing YNB, yeast extract, peptone and a suitable carbon source are commonly used for protein expression in *K. phaffii* shake flask cultivations but not bioireactors. Yeast extract and peptone present in these media stabilize secreted proteins, prevent or decrease proteolysis of secreted proteins and allow better growth and biomass accumulation (https://assets.thermofisher.com/TFS-Assets/LSG/manuals/easyselect_man.pdf). The recombinant protein induction in INDI-2 medium was performed by culturing cells in buffered medium containing 1.0% yeast extract, 2.0% peptone, 0.17% YNB (BD Biosciences, Cat: 233520) without ammonium sulfate, 0.5% urea, 100 mM potassium phosphate (pH 6.0), 0.4 mg/L biotin, 2.0% MSG, and 2% ethanol. The induction was carried out in a shake flask (culture to flask volume ratio 1:10) at 30 °C in an orbital shaker at 180 rpm with the addition of both inducers (MSG and ethanol) every 24 h. Food grade MSG used in this study is marketed online by Amazon, India as Ajinomoto.

### Generation of recombinant yeast and bacterial strains

**Fig. 1:**
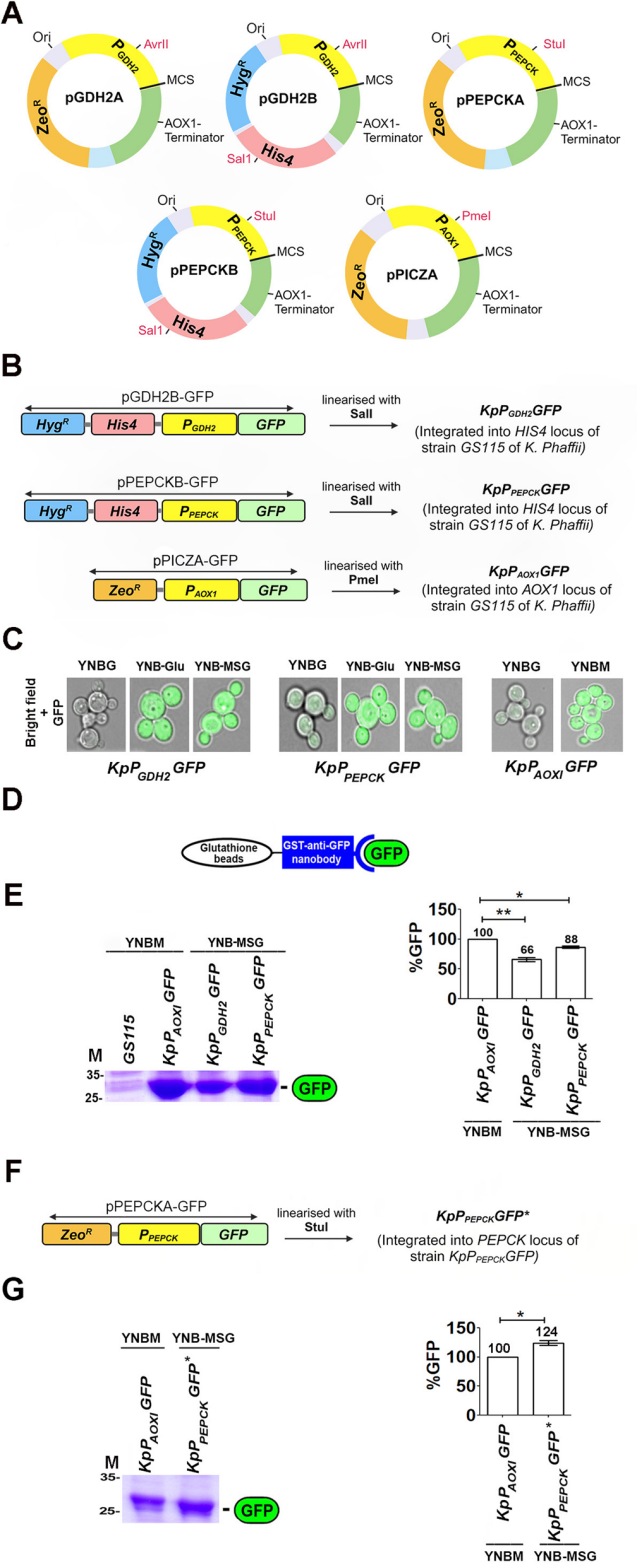
Construction of glutamate-inducible expression vectors and analysis of GFP expression in shake flask cultures of *K. phaffii*. **A** Key features of glutamate- and methanol-inducible expression vectors used in this study. Arrows indicate restriction sites used for linearization of the expression vectors for integration into the *K. phaffii* genome. Vector maps are provided in Additional file 1: Fig. S1. **B** Strategy for the generation of recombinant *K. phaffii* strains expressing GFP from *P*_*GDH2*_*, P*_*PEPCK*_ and *P*_*AOX1*_ (Table 1). The gene encoding GFP was cloned downstream of *P*_*GDH2*_*, P*_*PEPCK*_ and *P*_*AOX1*_ in expression vectors carrying different selection markers. Recombinant plasmids were linearized with SalI/Pme1 and transformed into *K. phaffii GS115* for integration at the *HIS4*/*AOXI* locus as indicated. **C** GFP expression profile from *P*_*AOX1*_*, P*_*GDH2*_ and *P*_*PEPCK*_ in cells cultured in YNB containing glycerol (YNBG), methanol (YNBM), glutamate (YNB-Glu) or MSG (YNB-MSG) by live cell confocal imaging. GFP expression was induced for 12 h. **D** Schematic representation of the strategy for purification of GFP from *K. phaffii* cell lysates by GST-tagged anti-GFP nanobody-mediated pull-down. **E** Comparative analysis of GFP expression from *P*_*AOX1*_,* P*_*GDH2*_ and *P*_*PEPCK*_ in cells cultured in YNBM (*P*_*AOX1*_) and YNB-MSG (*P*_*GDH2*_ and *P*_*PEPCK*_). GFP was purified from whole cell lysates of cells equivalent to 50 A_600_ units using glutathione-S-transferase (GST)-tagged anti-GFP nanobodies. GFP bound to GST-tagged anti-GFP nanobodies was subjected to SDS‒PAGE, and proteins were visualized by Coomassie Brilliant Blue R staining (left panel). GFP expression was induced for 24 h. M, protein molecular weight markers (kDa). Right panel, quantitation of GFP bands in the gel by densitometric scanning using ImageJ. MSG-inducible GFP expression from *P*_*GDH2*_ and *P*_*PEPCK*_ was normalized to methanol-inducible expression from *P*_*AOX1*_. **F** Strategy for the integration of *pPEPCKA-GFP* at the *HIS4* locus of *KpP*_*PEPCK*_*GFP* to generate *KpP*_*PEPCK*_GFP^*^, in which the *P*_*PEPCK*_*-GFP* expression cassette is integrated at two genomic loci (*PEPCK, HIS4*). **G** Analysis of GFP bound to GST-tagged, anti-GFP nanobodies by SDS‒PAGE. GFP was purified from whole cell lysates of cells equivalent to 20 A_600_ units. The gel was stained with Coomassie Brilliant Blue R (left panel). GFP expression was induced for 24 h. M, protein molecular weight markers (kDa). Right panel, quantification of GFP in the gel by densitometric scanning using ImageJ. Error bars in the graphs denote the mean ± S.D. from three biological replicates (n = 3), and the p value obtained from Student’s t test is mentioned on the bar of each figure: *P < 0.05; **P < 0.005; ***P < 0.0005; ns, not significant

**Fig. 2:**
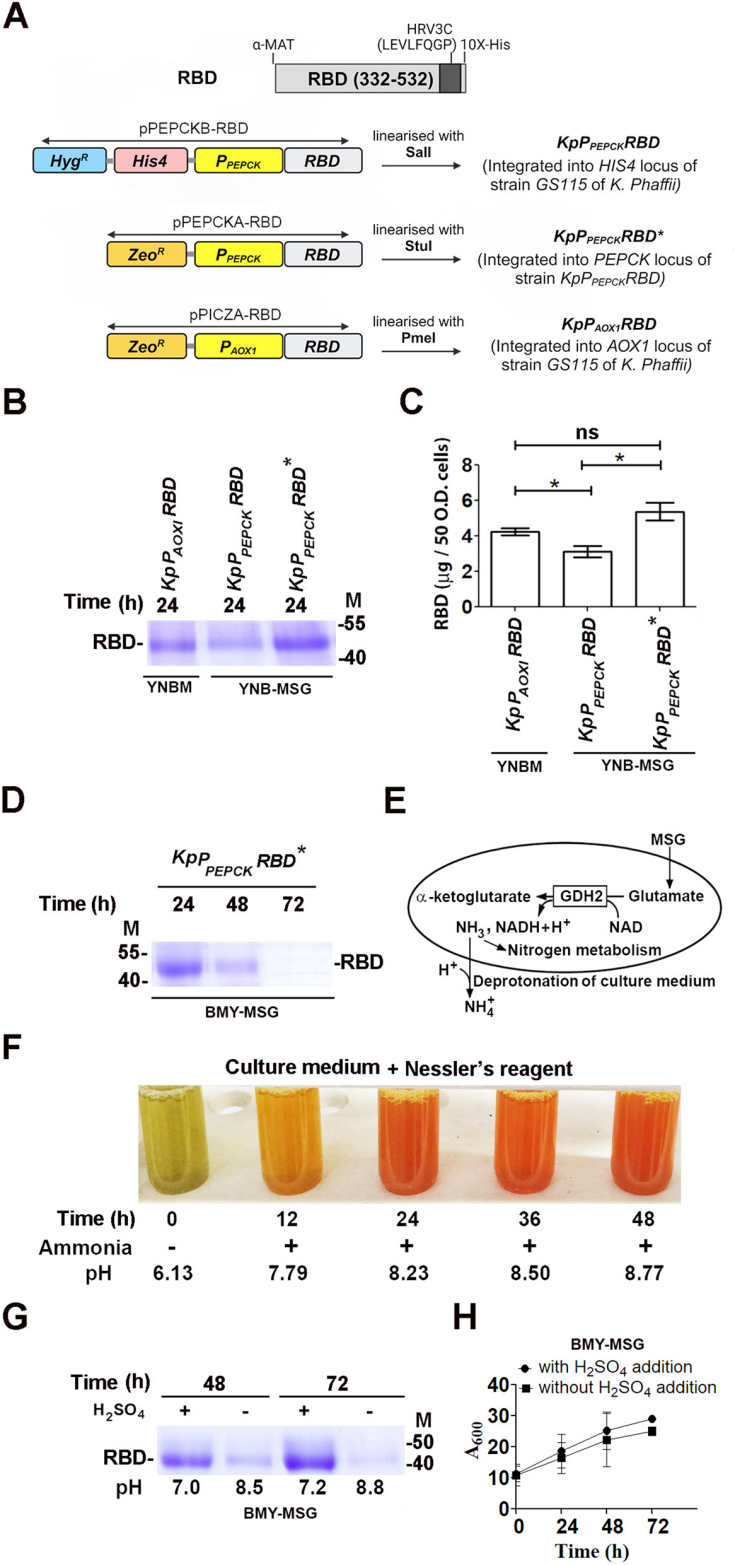
Analysis of the expression of the RBD from *P*_*PEPCK*_ and *P*_*AOX1*_ in shake flask cultures of *K. phaffii*. **A** Strategy for the generation of *KpP*_*AOX1*_*RBD* and *KpP*_*PEPCK*_*RBD* expressing His-tagged RBD from *P*_*AOX1*_ and *P*_*PEPCK,*_ respectively. *KpP*_*PEPCK*_*RBD* was further transformed with *pPEPCKA-RBD* to generate *KpP*_*PEPCK*_RBD^*^ (Table 1), in which the *P*_*PEPCK*_*-GFP* expression cassette is integrated at the *PEPCK* and *HIS4* loci of the genome. **B** SDS‒PAGE analysis of RBD secreted into medium by cells cultured for 24 h in YNBM or YNB-MSG as indicated. RBD was purified from the culture medium of cells equivalent to 50 A_600_ units. His-tagged RBD bound to Ni-agarose beads was subjected to SDS‒PAGE. M, molecular weight markers (kDa). **C** Quantification of RBD in the gel by densitometric scanning using ImageJ. Expression in *KpP*_*AOX1*_*RBD* was taken as 100%. Error bars in the graphs indicate the mean ± SD of 3 biological replicates. The p value was obtained from Student’s t test and is mentioned on the bar. ^*^p < 0.05; ns, not significant. **D** Protein profile of RBD purified from *KpP*_*PEPCK*_RBD^*^ cultured for 24 h, 48 h and 72 h in BMY-MSG as visualized by SDS‒PAGE. His tagged RBD present in the culture medium of cells equivalent to 50 A_600_ units was bound to Ni–NTA agarose beads and visualized by SDS‒PAGE. M, molecular weight markers (kDa). **E** Schematic diagram of ammonia production from the GDH2-catalyzed reaction and its efflux in the culture medium, resulting in alkalization. **F** Colorimetric detection of ammonia in the culture medium using Nessler’s reagent containing K_2_HgI_4_ and KOH. A reddish‐brown complex formed by the reaction of ammonia with iodide and mercury ions under alkaline conditions is detected spectrophotometrically by measuring absorbance at 420 nm. The pH of the culture medium measured at different time intervals is indicated. **G** Effect of maintenance of extracellular pH at ~ 7.0 by the addition of H_2_SO_4_ to BMY-MSG on RBD levels M, molecular weight markers (kDa). **H** Effect of H_2_SO_4_ addition on the growth of *KpP*_*PEPCK*_RBD^*^ cultured in BMY-MSG for up to 72 h. Data are the average of two independent experiments

Recombinant plasmids (Fig. 1A, B and Fig. 2A) were transformed into *Escherichia coli DH5α* strain by the CaCl_2_ method. *K. phaffii* strains used in this study are listed in Table 1. pPICZA and pPEPCKB/pPEPCKA plasmids (Fig. 1A) were used for methanol- and glutamate-inducible expression of target genes from *P*_*AOX1*_ and* P*_*PEPCK*_ respectively. The *AOX1* locus of *K. phaffii GS115* (*HIS4*^*−*^) was used for genomic integration of recombinant pPICZA as it is the only region in the vector having homology to *K. phaffii* genome. *HIS4* gene of pPEPCKB and histidine auxotrophy of GS115 were exploited for the genomic integration of recombinant pPEPCKB at *HIS4* locus of GS115 and selection of recombinants. The *PEPCK* locus of GS115 was used for integration of recombinant pPEPCKA into *K. phaffii* genome. Thus, by integrating the expression cassette at both *HIS4* and *P*_*PEPCK*_ loci, we were able to generate a *K. phaffii* strain in which the target gene cloned downstream of *P*_*PEPCK*_ is integrated at two different genomic loci. Yeast cells were transformed by electroporation (Gene Pulser, Bio-Rad, CA).

**Table 1:** Yeast strains used in this study

*K. phaffii* strain	Description	Source
*GS115*	*his4*	Ref. [38]
*Kp-P*_*AOX1*_*-GFP*	*GS115, his4 Zeo*^*r*^*::(pPICZA-P*_*AOX1*_*-GFP)*	This study
*Kp-P*_*GDH2*_*-GFP*	*GS115, his4*^+^,*Hyg*^*r*^*::(pGDH2B-P*_*GDH2*_*GFP)*	This study
*Kp-P*_*PEPCK*_*-GFP*	*GS115, his4*^+^,*Hyg*^*r*^*::(pPEPCKB-P*_*PEPCK*_*GFP)*	This study
*Kp-P*_*PEPCK*_-GFP^*^	*GS115, his4*^+^*,Hyg*^*r*^*,Zeo*^*r*^*::(pPEPCKB-P*_*PEPCK*_*-GFP, pPEPCKA-P*_*PEPCK*_*-GFP)*	This study
*Kp-P*_*AOX1*_*-RBD*	*GS115, his4 Zeo*^*r*^*::(pRBD)*	This study
*Kp-P*_*PEPCK*_*-RBD*	*GS115, his4*^+^*,Hyg*^*r*^*::(pPEPCKB-P*_*PEPCK*_*RBD)*	This study
*Kp-P*_*PEPCK*_-RBD^*^	*Kp-P*_*PEPCK*_*-RBD, Zeo*^*r*^*::(pPEPCKB-P*_*PEPCK*_*-RBD, pPEPCKA-P*_*PEPCK*_*-RBD)*	This study

### Antibodies and other reagents

Oligonucleotides were purchased from Sigma‒Aldrich (Bangalore, India). Mouse anti-GFP was purchased from Santa Cruz Biotechnology Inc. (Santa Cruz, CA) and mouse anti-His tag antibodies were purchased from Cell Signaling Technology (Denvers, MA). Restriction enzymes, NEB Hi-Fi builder (E2621L) and T4 DNA ligase were purchased from New England Biolabs (Ipswich, MA). DNA polymerases were purchased from GeNei (Bangalore, India) and Thermo Fisher Scientific (Waltham, MA).

### Generation of ***Kp-P***_***AOX1***_***-GFP, Kp-P***_***GDH2***_***-GFP and Kp-PPEPCK-GFP***

*Kp-P*_*AOX1*_*-GFP* was generated by PCR amplification of 714 bp of *GFP* ORF from *pREP41GFP* [27] using a primer pair containing overlapping regions of pPICZA suitable for cloning by Gibson assembly. The vector pPICZA was digested with Not1. The PCR-amplified GFP was cloned and inserted into the Not1 site of pPICZA using NEB-HiFi builder to generate *pPICZA-GFP.* For generation of *KpP*_*GDH2*_*GFP* and *Kp-P*_*PEPCK*_*-GFP*, GFP was amplified from *pREP41GFP* [27] using primer pairs containing overlapping regions of vectors pGDH2B and pPEPCKB, respectively, suitable for cloning by Gibson assembly. pGDH2B and pPEPCKB were digested with Not1. The PCR-amplified GFP was cloned and inserted into pGDH2B and pPEPCKB using NEB Hi-Fi Builder to generate *pGDH2B-GFP* and *pPEPCKB-GFP,* respectively. The Gibson assembly of all three clones was transformed into *E. coli DH5α* competent cells by the heat-shock method. The recombinant plasmids *pPICZA-GFP*, *pGDH2B-GFP* and *pPEPCKB-GFP* were linearized with Pme1, AvrII and Sal1, respectively, and transformed by electroporation into *GS115*. Recombinant clones were selected by plating on YPD-Zeocin (Zeocin, Invitrogen-R25001) and YPD hygromycin (Hygromycin, SRL 67317) plates. Clones expressing GFP were confirmed by checking GFP expression under a fluorescence microscope. Nucleotide sequences of primers will be provided on request.

### GST pull-down assay

*E. coli* cells (*BL21DE3*) expressing GST-tagged anti-GFP nanobody (Addgene plasmid #61838) [28] were suspended in 1X PBS (137 mM NaCl, 2.7 mM KCl, 100 mM Na_2_HPO_4_ and 2 mM KH_2_PO_4_, pH 7.4) containing 1 mM PMSF, 1 mM EDTA, 1 mM DTT, 10 mg/ml lysozyme and 1% Triton X-100 and kept on ice for 20 min. Cells were then sonicated, and cell lysates containing GST-tagged anti-GFP nanobodies were incubated with glutathione resin (G-Biosciences, U.S.A.) at 4 °C for 1 h. Nanobody-bound glutathione resins were harvested by brief centrifugation followed by washes with 1X PBS containing 1 mM PMSF. GST-tagged anti-GFP nanobody-bound glutathione resins were incubated with yeast (*GS115*) whole-cell protein lysate containing GFP expressing *P*_*AOX1*_*, P*_*GDH2*_ or *P*_*PEPCK*_ at 4 °C for 2–3 h. Post-interaction, resins were centrifuged briefly and washed at least thrice with cold 1X PBS. Bound proteins were resolved by SDS‒PAGE and visualized by Coomassie Brilliant Blue R-250 staining.

### Generation of*** Kp-PPEPCK-RBD and Kp-P***_***AOX1***_***-RBD***

For the generation of *Kp-P*_*PEPCK*_*-RBD*, coding SARSCoV2-RBD (RBD), *RBD* was amplified by PCR from pRBD [29]. The 10X-His-tagged RBD with an α-secretary signal was PCR amplified using a primer pair suitable for Gibson assembly at the Not1 site of pPEPCKB. Vector pPEPCKB was digested with Not1. The digested vector and RBD amplicon were subjected to Gibson assembly using NEB Hi-Fi Builder. The Gibson assembly mix was then transformed into *E. coli DH5α* competent cells using the heat-shock method. The recombinant plasmid (*pPEPCKB-RBD*) was linearized with SalI and transformed by electroporation into *GS115* for integration into the *His4* locus. Recombinant clones were selected by plating on YNBD His^−^ plates, and clones were confirmed by western blotting using an anti-His tag antibody. The primer sequence will be provided upon request. pRBD expressing RBD-10X-His under the *AOX1* promoter was a kind gift from Raghavan Varadarajan [29]. The vector designated as *pPICZA-RBD* was linearized by digestion with the Pme1 site and transformed by electroporation into *GS115* for integration into the *AOX1* locus. Recombinant clones were selected by plating on YPD Zeocin plates, and clones were confirmed by western blotting using an anti-His tag antibody.

### Generation of Kp-PPEPCK-GFP* and Kp-PPEPCK-RBD*

For the generation of *Kp-P*_*PEPCK*_-GFP*, 714 bp of *GFP* ORF was amplified from *pREP41GFP* using the primer pair containing the overlapping region of vector pPEPCKA for Gibson assembly. pPEPCKA was digested with Not1. The PCR-amplified GFP was cloned and inserted into pPEPCKA using NEB Hi-Fi Builder to generate *pPEPCKA-GFP*. The Gibson assembly mix was then transformed into *E. coli DH5α* competent cells. The *pPEPCKA-GFP* plasmid was linearized with Stu1 and transformed by electroporation into *Kp-P*_*PEPCK*_*-GFP* for integration into *PEPCK* locus. Recombinant clones were selected by plating on YNBD His^−^ Zeocin plates.

For the generation of *Kp-P*_*PEPCK*_-RBD*, the *RBD* was amplified from *pPEPCKB-RBD* using a primer pair suitable for Gibson assembly at the Not1 site of pPEPCKA. pPEPCKA was digested with Not1. The digested vector and RBD amplicon were subjected to Gibson assembly using NEB Hi-Fi Builder. The Gibson assembly mix was then transformed into *E. coli DH5α* competent cells using the heat-shock method. The recombinant plasmid (*pPEPCKA-RBD*) was linearized with Stu1 for integration into *PEPCK* locus of *Kp-P*_*PEPCK*_*-RBD* to generate *Kp-P*_*PEPCK*_-RBD*. Recombinant clones were selected by plating on YNBD His^−^ Zeocin plates. The primer sequence will be provided upon request. The yeast strains used in this study are listed in Table 1.

### Purification of His-tagged RBD

For the purification of His-tagged RBD secreted into the culture medium, cultures were harvested by centrifugation, and the culture medium was incubated with 1X PBS washed Ni–NTA agarose beads at 4 °C for 2 h. After interaction, RBD-bound beads were centrifuged briefly (2000 rpm for 2 min) and washed at least thrice with cold wash buffer containing 50 mM Tris (pH 8.0), 250 mM NaCl, 10% glycerol, 5 mM β-mercaptoethanol, 10 mM (PMSF) and 20 mM imidazole (pH 7.0). Bound proteins were then eluted by incubating the beads in ice-cold elution buffer containing 50 mM Tris (pH 8.0), 150 mM NaCl, 10% glycerol, 5 mM β-mercaptoethanol, and 300 mM imidazole (pH 7.0) for 1 h. Eluted proteins were quantified by performing a Bradford assay. The eluted protein was run on SDS‒PAGE and visualized by Coomassie Brilliant Blue R staining. The identity of the proteins was also confirmed by western blot analysis as described [4]

### Microscopy

Cells cultured in the respective media were washed with 1X PBS (137 mM NaCl; 2.7 mM KCl; 4.3 mM Na2HPO4; 1.47 mM KH2PO4. Adjust to a final pH of 7.4) once by centrifuging at 5000 rpm for 5 min. The washed cells were visualized under a fluorescence microscope (Olympus BX53) at 100X by direct florescence under a FITC filter. The images were captured and processed using Cell Sense Dimension software.

## Results

### MSG-inducible expression of GFP from ***P***_***GDH2***_*** and PPEPCK***

This study commenced with the construction of four synthetic glutamate-inducible expression vectors: *pGDH2A, pGDH2B, pPEPCKA* and *pPEPCKB* (Fig. 1A and Additional file 1: Fig. S1). GFP gene was cloned into *pGDH2B* and *pPEPCKB*, downstream of *P*_*GDH2*_ or *P*_*PEPCK*_, respectively, generating recombinant GFP expression vectors (Fig. 1B). These were linearized with SalI and integrated into *K. phaffii GS115* (*his*^*−*^) at the *HIS4* locus by electroporation (Fig. 1B). As a reference, *GFP* was also cloned into *pPICZA* downstream of *P*_*AOX1*_, linearized with Pme1 and integrated into *K. phaffii GS115* (*his*^*−*^) at the *AOX1* locus (Fig. 1A, B). Induction from *P*_*GDH2*_ and *P*_*PEPCK*_ in transformants was carried out using glutamate or monosodium glutamate (MSG), with the latter chosen for its solubility, safety, availability, and cost-effectiveness. GFP expression from *P*_*AOX1*_ was induced by methanol. Visualization under a fluorescence microscope confirmed GFP expression (Fig. 1C).

To compare GFP yields from *P*_*GDH2*_*, P*_*PEPCK*_ and *P*_*AOX1*_, cells were precultured in YPD for 24 h, transferred to yeast nitrogen base (YNB) with 0.5% ammonium sulfate and 2% methanol (YNBM) or 2% MSG (YNB-MSG) and cultured for 24 h in shake flasks. GFP was purified from whole cell lysates of cells equivalent to 50 OD_600_ units using glutathione-S-transferase (GST)-tagged anti-GFP nanobodies [28] and visualized by SDS‒PAGE (Fig. 1D, E). Densitometry scanning of protein bands revealed that the GFP yields from *P*_*GDH2*_ and *P*_*PEPCK*_ were 66% and 88%, respectively, of that from P_*AOXI*_ (Fig. 1E). While *P*_*PEPCK*_ yielded more GFP than *P*_*GDH2,*_ it fell short of the yield from *P*_*AOX1*_. To enhance the *P*_*PEPCK*_-driven GFP yield, *pPEPCKA-GFP* was generated and transformed into *KpP*_*PEPCK*_*GFP*. resulting in *KpP*_*PEPCK*_GFP^*^ with the *P*_*PEPCK*_-GFP expression cassette integrated at both the *HIS4* and *PEPCK* loci of the genome (Fig. 1F). Lysates from cells equivalent to 20 OD_600_ units were prepared, GFP was purified using anti-GFP nanobodies and visualized by SDS‒PAGE. Densitometric scanning indicated a 24% increase in GFP yield in *KpP*_*PEPCK*_GFP^*^ compared to *KpP*_*AOX1*_*GFP* (Fig. 1G).

### MSG-inducible expression of the SARS-CoV-2 virus receptor binding domain (RBD) from *PPEPCK*

To demonstrate the applicability of the glutamate-inducible expression system for vaccine antigen production, the SARS-CoV-2 virus receptor binding domain (RBD) was selected. RBD has been successfully expressed in mammalian cell lines as well as *K. phaffii* [29–35]. A synthetic gene encoding MATα secretory signal, amino acid residues 332–532 of RBD (Wuhan-Hu-1 strain), a C-terminal HRV-3C precision protease cleavage site and 10 × histidine tag, codon optimized for *K. phaffii* was amplified by PCR from pRBD [29], cloned downstream of *P*_*PEPCK*_. Recombinant strains *KpP*_*PEPCK*_*RBD* and *KpP*_*PEPCK*_RBD* were generated by integrating the expression cassette at *HIS4* alone and *HIS4* as well as *PEPCK* loci, respectively (Fig. 2A). For comparison, the *KpP*_*AOX1*_*RBD* strain was generated for methanol-inducible synthesis (Fig. 2A). After preculturing cells in YPD for 24 h, induction in YNB medium with MSG or methanol was carried out for 24 h. Cells equivalent to 50 OD_600_ units were centrifuged, His-tagged RBD was purified from the culture media by Ni–NTA chromatography, visualized on SDS polyacrylamide gel and quantified by densitometric scanning (Fig. 2B). The RBD yields from *KpP*_*PEPCK*_*RBD* and *KpP*_*PEPCK*_RBD^*^ were 74% and 127%, respectively, of that from *KpP*_*AOX1*_*RBD* (Fig. 2C).

The study then explored RBD expression up to 72 h from *P*_*PEPCK*_ by culturing *KpP*_*PEPCK*_RBD^*^ in buffered complex MSG medium (BMY-MSG) consisting of 1% yeast extract, 2% peptone, 100 mM potassium phosphate pH 6.0, 0.17% YNB with ammonium sulfate and 0.4 mg/L biotin. BMY-MSG is similar to buffered modified methanol (BMMY) medium used for inducing protein expression from *P*_*AOXI*_ except for substitution of methanol with 2% MSG. After preculturing *KpP*_*PEPCK*_RBD^*^ in YPD for 24 h, induction in BMY-MSG was carried out for 24 h, 48 h and 72 h. MSG was replenished every 24 h. Unexpectedly, the RBD yield decreased after 24 h of induction (Fig. 2D). The probable cause for the same could be glutamate metabolism-induced ammonia accumulation leading to alkalinization of the culture medium [36] (Fig. 2E). This was demonstrated by culturing *K. phaffii* in YP-MSG containing 1% yeast extract, 2% peptone and 2% MSG instead of BMY-MSG to avoid interference by ammonium ions in YNB and detecting ammonia in the culture medium colorimetrically using Nessler’s reagent [37]. A time-dependent increase in brown precipitate formation was observed due to the reaction of Nessler’s reagent with ammonia, confirming that presence of ammonia in the medium as the cause for the alkalization of the culture medium (Fig. 2F). It is likely that an increase in pH beyond the isoelectric point of RBD (8.1) reduces solubility, resulting in precipitation and decrease in RBD yield. Periodic addition of H_2_SO_4_ improved RBD levels (Fig. 2G), but sustained growth was not observed (Fig. 2H), indicating that BMY-MSG is not suitable for long-term shake flask cultivation.

### Optimization of culture conditions for increasing recombinant protein yield from *PPEPCK*

**Fig. 3:**
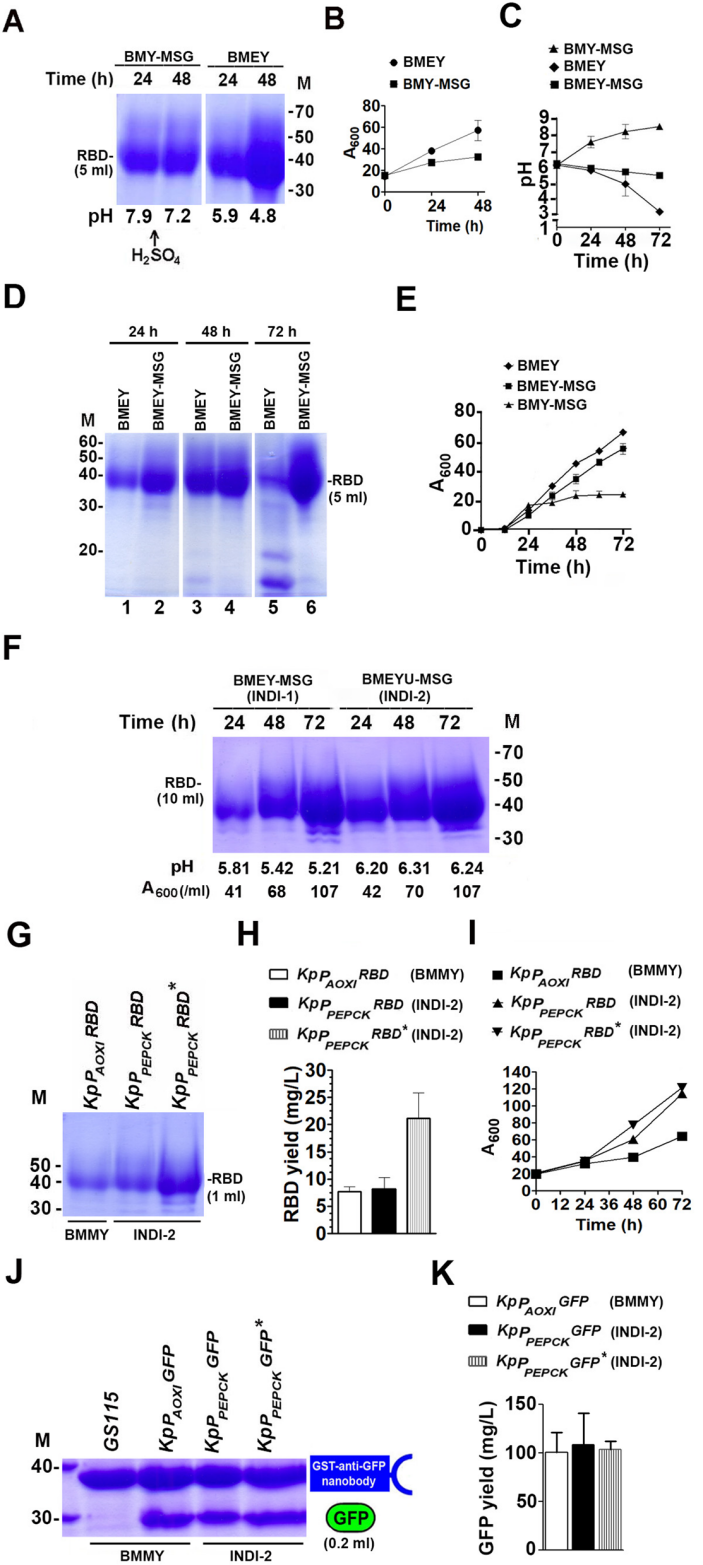
Optimization of induction medium for improving recombinant protein yield from* P*_*PEPCK*_. **A** Visualization of RBD secreted into medium by *KpP*_*PEPCK*_RBD^*^ cultured in BMY-MSG and BMEY for 24 h and 48 h by SDS‒PAGE. His-tagged RBD from 5 ml culture medium was bound to Ni-agarose beads and analyzed by SDS‒PAGE. The pH of the culture medium after 24 h and 48 h is indicated. H_2_SO_4_ was added to BMY-MSG after 24 h of induction to maintain pH < 7.5. M, molecular weight markers (kDa). **B** Analysis of the growth of *KpP*_*PEPCK*_RBD^*^ cultured in BMY-MSG and BMEY. Data are the average of two independent experiments. **C** Extracellular pH measured at different time intervals when *KpP*_*PEPCK*_RBD^*^ was cultured in BMY-MSG, BMEY and BMEY-MSG. **D** Visualization of RBD secreted into 5 ml of medium by *KpP*_*PEPCK*_RBD^*^ cultured in BMEY and BMEY-MSG for 24 h, 48 h and 72 h by SDS‒PAGE. His-tagged RBD bound to Ni-agarose beads was analyzed by SDS‒PAGE. M, molecular weight markers (kDa). **E** Analysis of the growth of *KpP*_*PEPCK*_RBD^*^ cultured in BMEY, BMEY-MSG and BMY-MSG. Data are the average of two independent experiments. **F** Visualization of RBD secreted into 10 ml of medium by *KpP*_*PEPCK*_RBD^*^ cultured in BMEY-MSG (INDI-1) and BMEYU-MSG (INDI-2) for 24 h, 48 h and 72 h by SDS‒PAGE. His-tagged RBD bound to Ni-agarose beads was analyzed by SDS‒PAGE. M, molecular weight markers (kDa). The growth of cells measured by absorbance at A_600_ as well as the pH of the culture medium are indicated. M, molecular weight markers (kDa). **G** Comparison of RBD yield from *KpP*_*AOX1*_*RBD*, *KpP*_*PEPCK*_*RBD and KpP*_*PEPCK*_RBD^*^ cultured for 72 h in BMMY or INDI-2 as indicated. One milliliter of culture medium was incubated with Ni-agarose beads, and RBD bound to the beads was eluted, estimated using Bradford reagent and examined by SDS‒PAGE. M, molecular weight markers (kDa). **H** Quantification of data presented in **G**. RBD was estimated using Bradford reagent from a standard curve generated from a known concentration of bovine serum albumin. Data are the average from three independent experiments (n = 3). Error bars in the graphs denote the mean ± S.D. Culture media are shown in parentheses. **I** Analysis of the growth of *KpP*_*AOX1*_*RBD*, *KpP*_*PEPCK*_*RBD* and *KpP*_*PEPCK*_RBD^*^. Culture media are shown in parentheses. Data are the average from three independent experiments (n = 3). **J** Comparison of GFP yield from *KpP*_*AOX1*_*GFP*, *KpP*_*PEPCK*_*GFP and KpP*_*PEPCK*_GFP^*^ cultured for 72 h in BMMY or INDI-2 as indicated. GFP was purified from whole cell lysates of 0.2 ml cells using glutathione-S-transferase (GST)-tagged anti-GFP nanobodies. GFP bound to GST-tagged anti-GFP nanobodies was visualized by SDS‒PAGE. M, molecular weight markers (kDa). **K** Quantitation of GFP bands in the gel by densitometric scanning using ImageJ. Data are the average from three independent experiments (n = 3). Error bars in the graphs denote the mean ± S.D. The numbers in parentheses indicate the volumes of culture used for purification of the recombinant protein (RBD/GFP). In all experiments, the carbon source was replenished every 24 h

In view of the poor growth and low RBD yield from cells cultured in BMY-MSG, we examined growth, extracellular pH and RBD production in *KpP*_*PEPCK*_RBD^*^ cultured for up to 48 h in buffered modified ethanol medium (BMEY) containing 2% ethanol instead of MSG since ethanol also induces PEPCK synthesis in cells cultured in YNBE [4]. The RBD yield in cells cultured in BMEY was either comparable (24 h) or higher (48 h) than that in cells cultured in BMY-MSG (Fig. 3A). This was surprising since ethanol is not as good an inducer of PEPCK as glutamate in cells cultured in YNB media [4]. Further, a decrease rather than an increase in extracellular pH was observed when cells were cultured in BMEY obviating the need for H_2_SO_4_ addition to prevent rise in extracellular pH (Fig. 3A). Growth of cells in BMEY was better than that in BMY-MSG (Fig. 3B), suggesting that an increase in RBD yield in the former may be due to the generation of higher biomass during ethanol metabolism than during glutamate metabolism. However, a steady decline in extracellular pH reaching 3.2 after 72 h of induction (Fig. 3C) resulted in a drastic decrease in RBD yield (Fig. 3D, lane 5). It is likely that protein unfolding and exposure of hydrophobic regions of RBD at low pH may reduce solubility resulting in aggregation and low yield. Thus, neither BMY-MSG nor BMEY served as a good induction medium for high yield recombinant protein production from *P*_*PEPCK*_ in shake flask cultivations.

The increase and decrease in extracellular pH observed when *K. phaffii* was cultured in BMY-MSG and BMEY, respectively, led us to investigate whether cofeeding ethanol and MSG can provide optimum growth conditions and high RBD yield. When *KpP*_*PEPCK*_RBD^*^ was cultured in BMEY-MSG (containing 2% ethanol as well as 2% MSG), the extracellular pH was maintained between 5.8 and 6.0 (Fig. 3C), and the RBD yield as well as growth improved significantly (Fig. 3D, E). Preculturing *KpP*_*PEPCK*_RBD^*^ up to an A_600_ of 20–25/ml followed by induction in BMEY-MSG for 72 h resulted in high biomass (A_600_ = 110/ml) (Fig. 3E), and maintenance of extracellular pH between 5.8 and 5.3 throughout the induction period (Fig. 3F). Substitution of ammonium sulfate in BMEY-MSG with 0.5% urea (BMEYU-MSG) resulted in maintenance of extracellular pH between 6.0 and 6.5, further improving RBD yield (Fig. 3F). Based on these results, BMEY-MSG (INDI-1) and BMEYU-MSG (INDI-2) are recommended as optimal induction media for glutamate-inducible recombinant protein production from *P*_*PEPCK*_ in shake flask cultivations.

After optimization, the RBD yields from *KpP*_*AOX1*_*RBD* in BMMY were compared to those from *KpP*_*PEPCK*_*RBD* and *KpP*_*PEPCK*_RBD^*^ in INDI-2 after 72 h of induction. The RBD yields were 10 ± 2 mg/L, 10 ± 2 mg/L and 25 ± 2 mg/L, respectively (Fig. 3G, H). The higher RBD yields from *P*_*PEPCK*_ were attributed to increased biomass generated in INDI-1/INDI-2 (Fig. 3I). GFP yields under similar culture conditions were comparable (100 ± 5 mg/L) for *KpP*_*AOX1*_*GFP*, *KpP*_*PEPCK*_*GFP and KpP*_*PEPCK*_GFP^*^ (Fig. 3J, K). Thus, ethanol-MSG cofeeding strategy generated high biomass during the induction phase, resulting in recombinant protein yield from *P*_*PEPCK*_ equal to or surpassing that from the methanol-inducible *P*_*AOX1*_.

**Fig. 4:**
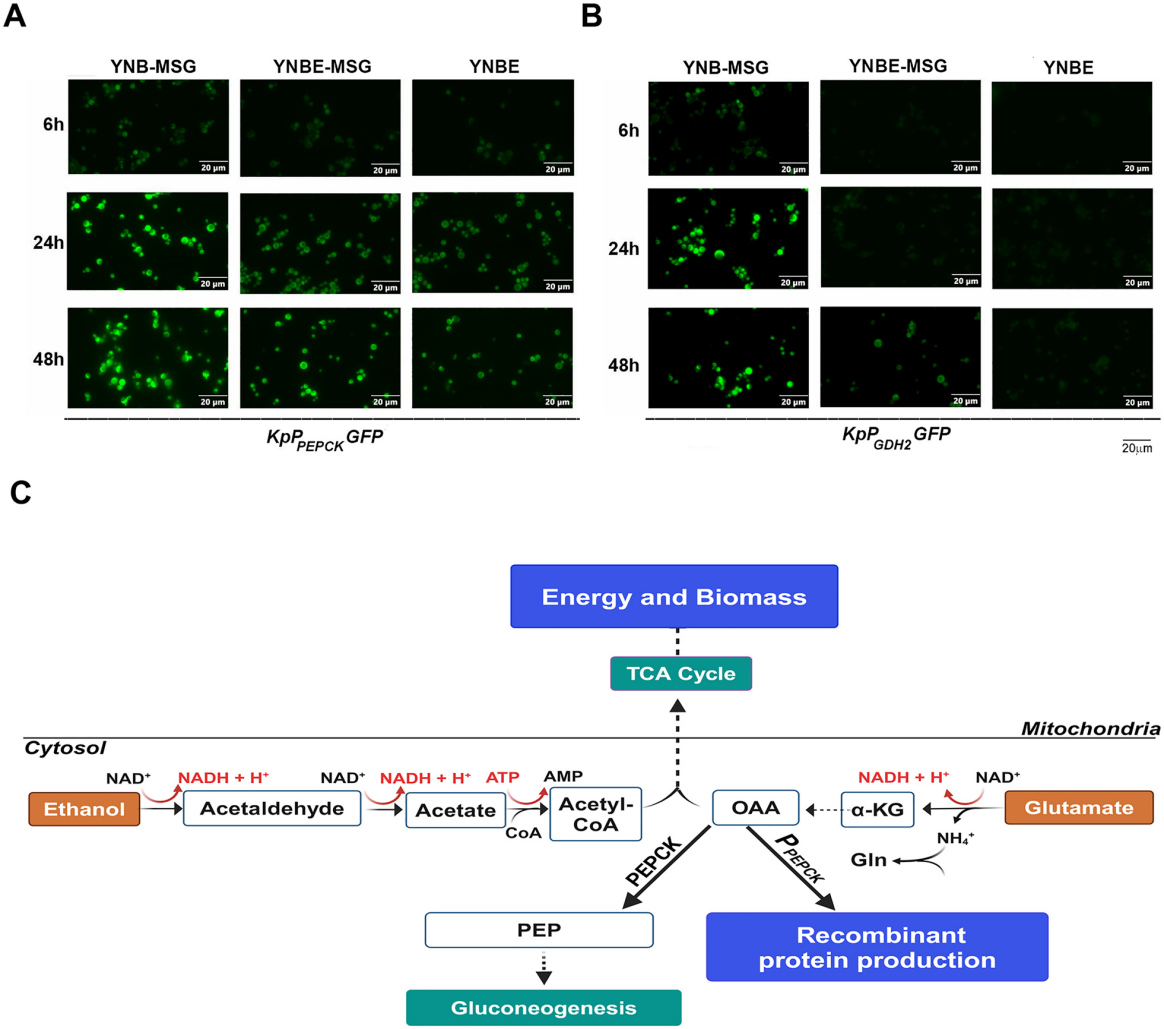
Analysis of GFP expression in *KpP*_*PEPCK*_*GFP* (**A**)and *KpP*_*GDH2*_*GFP* (**B**) cultured in YNB-MSG, YNBE and YNBE-MSG for 6 h, 24 h and 48 h by live cell imaging under a fluorescence microscope. See text for details. **C** Schematic representation of biochemical events taking place during ethanol-MSG co-feeding. Ammonia generated during the conversion of glutamate to α-ketoglutarate (α-KG) by GDH2 combines with H^+^ generated during ethanol metabolism and the resulting NH4^+^ is utilized for biochemical reactions such as glutamine biosynthesis catalyzed by glutamine synthetase. Oxaloacetate (OAA) synthesized from α-KG induces synthesis of PEPCK and the recombinant proteins via the *P*_*PEPCK*_-translational regulatory circuit [4]. PEPCK converts OAA to phosphoenolpuruvate (PEP) which is utilized in gluconeogenesis. Acetyl Co-A and OAA generated from ethanol and glutamate metabolism enter mitochondrial TCA cycle and contribute to ATP which is utilized for growth and generation of biomass

To understand *P*_*PEPCK*_ and *P*_*GDH2*_ activity during ethanol-MSG cofeeding, *KpP*_*PEPCK*_*GFP* and *KpP*_*GDH2*_*GFP* were cultured in YNB-MSG, YNBE-MSG and YNBE for 48 h. GFP expression was visualized by confocal microscopy at 6 h, 24 h and 48 h. At 24 h, GFP expression pattern in *KpP*_*PEPCK*_*GFP* cultured in YNBE + MSG was similar to that in YNBE suggesting ethanol rather than MSG is likely to be the inducer at this time point. At the same time, there was no observable expression of GFP from *P*_*GDH2*_ in *KpP*_*GDH2*_*GFP* cultured in YNBE + MSG supporting the hypothesis that ethanol utilization precedes MSG (Fig. 4A, B). At 48 h, GFP expression in *KpP*_*PEPCK*_*GFP* cultured in YNBE + MSG is similar to that in YNB-MSG suggesting MSG is likely to be the inducer which was further confirmed by the GFP expression pattern observed in *KpP*_*GDH2*_*GFP* in YNBE + MSG medium (Fig. 4A, B). These results indicate that ethanol is the preferred source of carbon during mixed feeding and MSG is unable to induce *P*_*PEPCK*_ in the presence of ethanol. During ethanol-MSG mixed feeding, ethanol is utilized as the carbon source and glutamate primarily contributes to glutamine synthesis and nitrogen metabolism. Generation of oxaloacetate and acetyl Co-A from glutamate and ethanol metabolism respectively contribute to generation of ATP and biomass via their participation in TCA cycle. We propose that ammonia and H^+^ generated during ethanol-MSG mixed feeding are utilized intracellularly for metabolic reactions including glutamine synthesis, not released into the medium and thus do not contribute to variations in extracellular pH observed during ethanol or MSG feeding. These events are schematically depicted in Fig. 4C.

## Discussion

Methanol-inducible recombinant protein production from *K. phaffii P*_*AOX1*_ in shake flasks is carried out by culturing cells in a medium containing a repressible carbon source upto cell density equivalent to A_600_ of 15–20/ml and then transferring to BMMY for inducing recombinant protein production for at least 72 h. We examined whether similar culture conditions can be adapted for MSG-inducible protein production from *K. phaffii P*_*PEPCK.*_ BMY-MSG was used as induction medium by substituting methanol in BMMY with MSG. Initial results were not satisfactory since an increase in extracellular pH and poor growth of cells in BMY-MSG resulted in low RBD yield. Several rounds of media optimization culminated in ethanol-MSG mixed feeding and development of a new induction medium named INDI-1 which supported high cell density during the induction period of 72 h resulting in high recombinant protein yield from *K. phaffii P*_*PEPCK*_ in shake flask cultures. Substitution of ammonium sulphate in INDI-1 with urea (INDI-2) resulted in better maintenance of extracellular pH, further improving recombinant protein yield. Analysis of growth as well as *GDH2* and *PEPCK* promoter activity suggest that generation of high biomass during ethanol-MSG co-utilization contributes for the high recombinant protein yield. We recommend either INDI-1 or INDI-2 as the induction medium for recombinant protein production from *P*_*PEPCK*_. GFP and RBD were used as examples for MSG-inducible intracellular and extracellular production of recombinant proteins, respectively, from *P*_*PEPCK*_ in *K. phaffii*. The best results were obtained with INDI-2, when protein expression from *P*_*PEPCK*_ equaled and even surpassed that from *P*_*AOX1*_. Multicopy genomic integration of target genes further improves recombinant protein yield as evident from higher RBD yield in *KpP*_*PEPCK*_GFP^*^ than that in *KpP*_*PEPCK*_*GFP.* Co-feeding ethanol and MSG resulted in not only maintenance of extracellular pH between 6.0 and 6.5 during the induction phase but also generated biomass almost as high as that generated by culturing cells in YPD or buffered glycerol complex medium (BMGY). Thus, *P*_*PEPCK*_ can be used for constitutive expression of proteins by directly inoculating cells in INDI-1/INDI-2 medium. This is recommended for production of those recombinant proteins where decoupling cell growth and product formation is not an absolute requirement. Synthesis of PEPCK is regulated at the translational level [4] and thus the glutamate-inducible expression system differs from other inducible *K. phaffii* inducible expression systems described till date which are primarily based on transcriptional activation of target genes. This is the first example of exploitation of a glutamate-inducible translational regulatory circuit for recombinant protein production in any yeast species.

Shake flasks are commonly used for production of recombinant proteins at the laboratory scale due to their very simple setup and operation. However, key limitations of shake flask cultivation include low aeration capacity and the batch mode of cultivation. The yield of recombinant proteins in shake flasks is usually much lower than that in bioreactors, where high oxygen transfer rates can result in robust growth, resulting in high recombinant protein yield. While developing a new expression system, optimizing culture conditions in shake flask cultivations is essential before its use in bioreactors.

## Conclusions

We report a novel glutamate-inducible protein expression system in the yeast *Komagatella phaffi*. We recommend two different induction media (INDI-1, INDI-2) for recombinant protein production from *K. phaffii P*_*PEPCK*_ in shake flask cultivations. INDI-1 consists of 1.0% yeast extract, 2.0% peptone, 0.17% YNB with ammonium sulfate, 100 mM potassium phosphate (pH 6.0), 0.4 mg/L biotin, 2.0% MSG, and 2% ethanol. In INDI-2, ammonium sulphate is substituted with 0.5% urea. Cell density of 100–120 A_600_ units/ml was achieved after 72 h of induction in shake flask cultivations, resulting in recombinant protein yield from *P*_*PEPCK*_ that is comparable or even higher than that from* P*_*AOX1*_. Optimization of culture conditions for MSG-inducible production of intracellular and secretory proteins in INDI-1/INDI-2 medium in this study sets the stage for industrial scale recombinant protein production from *K. phaffii P*_*PEPCK*_ in bioreactors.

### Supplementary Information

**Additional file 1: Fig. S1.** Maps of glutamate-inducible vectors (pGDH2A, pGDH2B, pPEPCKA, pPEPCKB) described in the study.

## Data Availability

The data that support the findings of this study are available from the corresponding author upon reasonable request.

## Abbreviations

YNB: Yeast nitrogen base
BMY: Buffered modified medium
MSG: Monosodium glutamate
GFP: Green fluorescent protein
RBD: Receptor binding domain of SARS Co-V-2 spike protein
GDH2: Glutamate dehydrogenase 2
PEPCK: Phosphoenolpyruvate carboxykinase
