# Supplementary material for: Unlocking Nature’s Toolbox: glutamate-inducible recombinant protein production from the Komagatella phaffii PEPCK promoter
Source: Microb Cell Fact. 2024 Feb 24;23:66. doi: 10.1186/s12934-024-02340-1 (PMC10893637; doi:10.1186/s12934-024-02340-1)
Supplement: Supplementary file 1 — Additional file 1: Fig. S1. Maps of glutamate-inducible vectors (pGDH2A, pGDH2B, pPEPCKA, pPEPCKB) described in the study. [file 12934_2024_2340_MOESM1_ESM.pdf]

Glutamate-inducible recombinant protein production from *Komagataella phaffii* PEPCK promoter

Neetu Rajak, Trishna Dey, Yash Sharma, Vedanth Bellad and Pundi N Rangarajan

Additional file 1

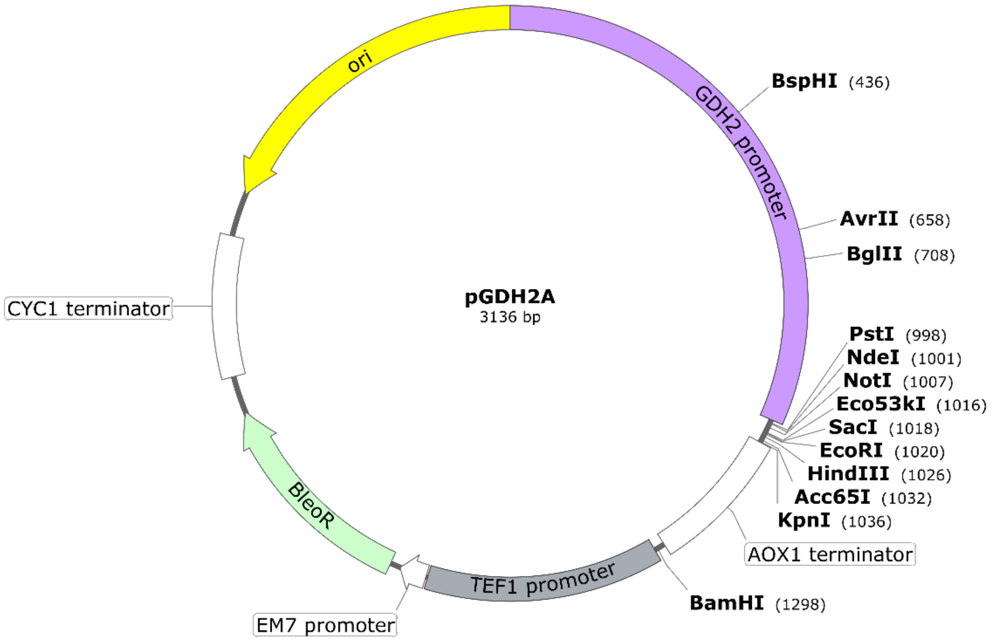

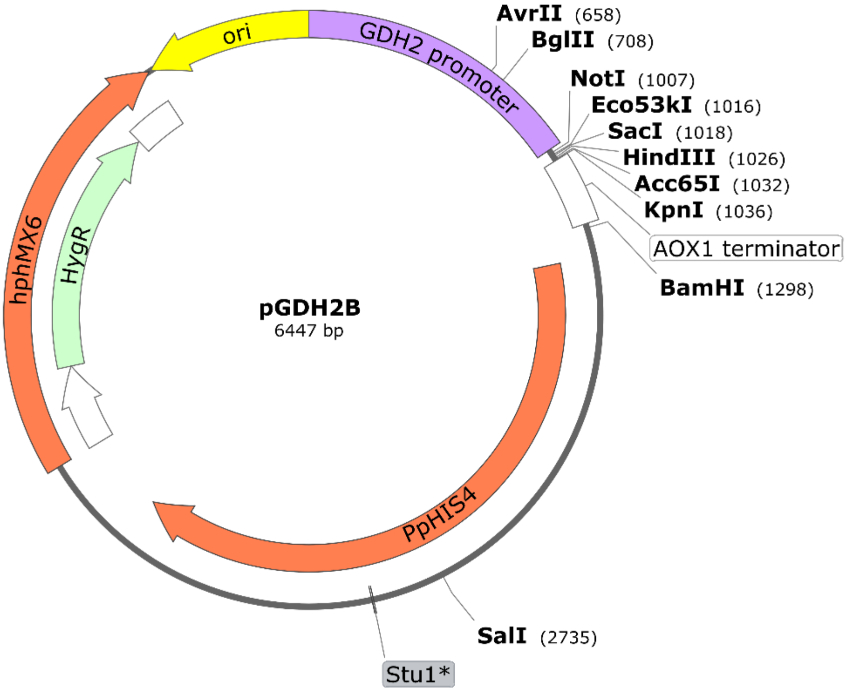

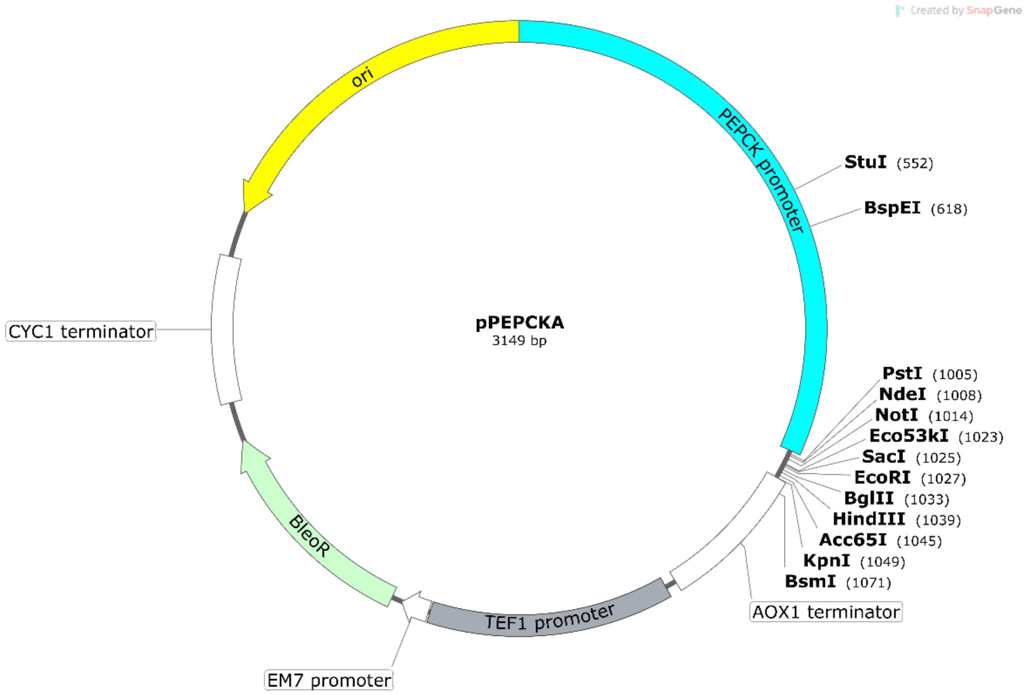

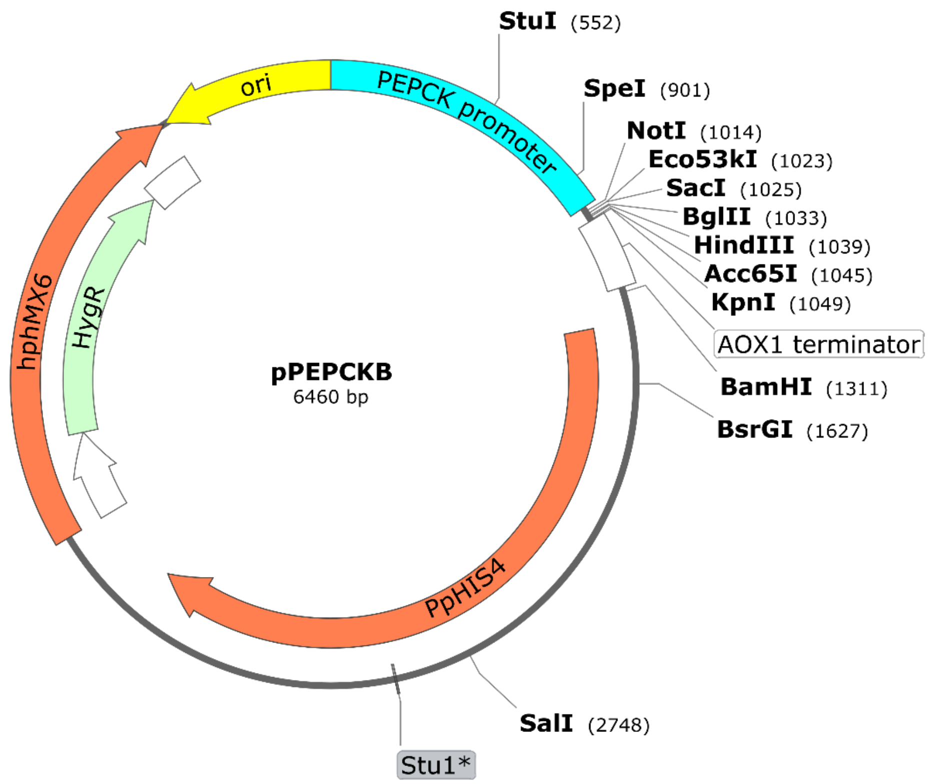

Additional file 1. Fig. S1. Maps of glutamate-inducible vectors (pGDH2A, pGDH2B, pPEPCKA, pPEPCKB) described in the study. Snapgene versions with complete nucleotide sequence of the vectors will be provided on request.
